# Genome Sequence of a Fowl Adenovirus Serotype 4 Strain Lethal to Chickens, Isolated from China

**DOI:** 10.1128/genomeA.00140-16

**Published:** 2016-03-24

**Authors:** Lintao Li, Ling Luo, Qingping Luo, Tengfei Zhang, Kang Zhao, Honglin Wang, Rongrong Zhang, Qin Lu, Zishu Pan, Huabin Shao, Wanpo Zhang, Guoyuan Wen

**Affiliations:** a Institute of Animal Husbandry and Veterinary Sciences, Hubei Academy of Agricultural Sciences, Wuhan, China; b College of Veterinary Medicine, Huazhong Agricultural University, Wuhan, China; c Hubei Key Laboratory of Animal Embryo and Molecular Breeding, Wuhan, China; d College of Life Sciences, Wuhan University, Wuhan, China

## Abstract

We report here the complete genome sequence of virulent fowl adenovirus serotype 4 strain HB1510, isolated from a diseased chicken with hydropericardium-hepatitis syndrome in Hubei, China, in 2015. The viral genome is 43,721 bp long, and sequence analysis showed an 11-amino-acid deletion in open reading frame 29 (ORF29).

## GENOME ANNOUNCEMENT

Hydropericardium-hepatitis syndrome (HHS) is a highly infectious disease in chickens, typically characterized by an accumulation of transparent or straw-colored fluid in the pericardial sac and hepatitis, with a high mortality rate of 30 to 70% (1). An outbreak of HHS was reported first in 1987 in Pakistan (2). After that, outbreaks have been documented in many countries, such as the United States, India, and South Korea, and result in considerable economic loss (3–5). The main causative agent of HHS is virulent fowl adenovirus (FAdV) serotype 4 (6), which belongs to family *Adenoviridae* and genus *Aviadenovirus*. However, the clinical cases of HHS and isolation of virulent FAdV-4 have rarely been reported in China.

In 2015, HHS emerged in many provinces of China and caused extensive epidemics in broiler and layer chickens. Most cases were observed in chickens of 2 to 4 weeks of age. The mortality during this outbreak was nearly 40%, up to 90% in severe cases. In a diseased chicken farm in Hubei Province, we collected liver and heart samples from a diseased chicken and isolated the virus in 9-day-old specific-pathogen-free (SPF) chicken embryos via the allantoic cavity route. An FAdV strain, HB1510, was isolated and subtyped as FAdV-4 by nucleotide sequence analysis of the hexon gene (7). In an animal experiment, 7 of 10 (70%) 14-day-old SPF chickens died within 4 days of being inoculated intramuscularly with 10^4.0^ 50% egg infective dose (EID_50_) of the HB1510 virus.

For the whole-genome sequencing, nine pairs of primers were designed to PCR amplify the different genomic fragments covering the entire genome of the HB1510 virus. The nine overlapping PCR products were purified and sequenced in both directions (Sangon, Shanghai, China). The sequences were compiled and edited using the SeqMan program (Lasergene) to produce the whole-genome sequence of strain HB1510. The complete genome of HB1510 virus was 43,721 bp in length, with 54.9% G+C content. The HB1510 virus had a typical genome organization of FAdV-4, with two fiber genes (fiber-1 and fiber-2). Both of its inverted terminal repeat (ITR) sequences were 56 bp long. Compared to the genome sequences of other published FAdV-4 strains, there was 98.3 to 99.9% homology at the nucleotide level. The HB1510 strain was most matched to the JSJ13 strain, with 28.6% mortality, isolated from China in 2013 (7). However, sequence alignment showed that the genome of HB1510 had an 11-amino-acid deletion (RPPPMITPLYT) at position 23 of open reading frame 29 (ORF29), which was a distinctive difference from the JSJ13 strain. Furthermore, the penton protein was highly conserved, with amino acid homology ranging from 98.7 to 100.0%.

Overall, the genome sequence of the virulent FAdV-4 strain HB1510 will profit aid further investigation on the epidemiology and evolution of FAdV-4 and may help explore the molecular mechanism of the viral pathogenesis.

### Nucleotide sequence accession number. 

The complete genome sequence of the virulent FAdV-4 strain HB1510 has been deposited to GenBank under the accession no. KU587519.
